# Non-bacterial Thrombotic Endocarditis in a Patient With Anorexia Nervosa: A Case Report

**DOI:** 10.7759/cureus.86378

**Published:** 2025-06-19

**Authors:** Tooba Iqbal, Musab Asif, Farzeen Saghir, Arslan Mubarik, Ayma Saleem

**Affiliations:** 1 Psychiatry and Behavioral Sciences, Nishtar Medical University, Multan, PAK; 2 Cardiology, Nishtar Medical University, Multan, PAK; 3 Medicine, Nishtar Medical University, Multan, PAK

**Keywords:** anorexia nervosa, cardiac complications of anorexia, eating disorder complications, hypercoagulability, hypoalbuminemia, medical-psychiatric interface, multidisciplinary management, non-bacterial thrombotic endocarditis, psychiatric complications, severe malnutrition

## Abstract

Non-bacterial thrombotic endocarditis (NBTE) is a rare complication typically seen in association with malignancies, autoimmune diseases, and hypercoagulable states. Its occurrence in the context of severe malnutrition, particularly in anorexia nervosa, is extremely uncommon. This case describes a 28-year-old woman with a long-standing history of anorexia nervosa (restrictive subtype), who presented with fatigue, dizziness, and chest discomfort. Clinical examination revealed a systolic murmur, and echocardiography identified sterile vegetations on the mitral valve. Blood cultures were negative, and further evaluation ruled out malignancy and autoimmune disease, leading to a diagnosis of NBTE likely precipitated by profound malnutrition. The patient was managed with therapeutic anticoagulation, cautious nutritional rehabilitation, and intensive psychiatric support. Over a four-week hospitalization, she showed significant improvement in nutritional status and resolution of vegetations on follow-up imaging. This case highlights the need to consider NBTE in severely malnourished individuals and underscores the importance of a multidisciplinary approach to address both the medical and psychiatric dimensions of anorexia nervosa.

## Introduction

Non-bacterial thrombotic endocarditis (NBTE), also referred to as marantic endocarditis, is a rare condition characterized by the formation of sterile fibrin and platelet vegetations on heart valves [1]. It is most commonly associated with hypercoagulable states, particularly in individuals with malignancies, autoimmune diseases, and systemic infections [2]. NBTE has been identified in approximately 1-1.6% of unselected adult autopsy series and in up to 9% of patients with advanced malignancies, although clinically recognized cases remain exceedingly rare (<1%). However, its occurrence in individuals suffering from severe malnutrition, such as those diagnosed with anorexia nervosa, is rarely documented. The case of a 28-year-old woman with a history of anorexia nervosa (restrictive subtype), who developed NBTE due to the severe metabolic and hemodynamic consequences of starvation, illustrates this rare but serious complication. This case report provides an in-depth analysis of the pathophysiology of NBTE in malnourished individuals, describes the diagnostic process, and outlines the comprehensive treatment approach that facilitated the patient’s recovery.

## Case presentation

Patient presentation

A 28-year-old woman presented to the emergency department with progressive shortness of breath, generalized weakness, and intermittent chest discomfort persisting for one week. She reported a significant decline in energy levels over the past three months, experiencing frequent dizziness and near-syncope, which coincided with a marked reduction in her food intake due to an intensifying fear of weight gain. Her medical history revealed a diagnosis of anorexia nervosa at the age of 18, but she had never engaged in formal psychiatric or nutritional treatment. There was no personal or family history of thromboembolic events, hypertension, diabetes, or cardiovascular disease. Her psychosocial background indicated a perfectionistic personality, severe body image disturbance, and overwhelming anxiety related to eating. Over the previous six months, she had lost approximately 8 kg, resulting in a weight of 34 kg and a dangerously low BMI of 12.5 kg/m².

Clinical examination

On physical examination, she appeared significantly undernourished and frail, with dry mucous membranes and lanugo hair on her extremities, signs consistent with chronic malnutrition. Her vital signs showed a heart rate of 110 bpm, low blood pressure (90/60 mmHg), and an oxygen saturation of 96% on room air. She was afebrile and did not exhibit any signs of respiratory distress. Cardiac auscultation revealed a faint grade 2/6 systolic murmur at the cardiac apex, without radiation. There were no peripheral stigmata of infective endocarditis, such as Janeway lesions, Osler nodes, or splinter hemorrhages. Peripheral pulses were weak but present. Her lungs were clear on auscultation, and the abdominal examination was unremarkable. Neurologically, she was alert and oriented, without any focal deficits. Given her medical history and the detection of a new murmur, differential diagnoses included anemia-related functional murmurs, pericardial effusion due to malnutrition, and potential endocarditis, either infective or non-infective.

Diagnostic workup

**Figure 1:**
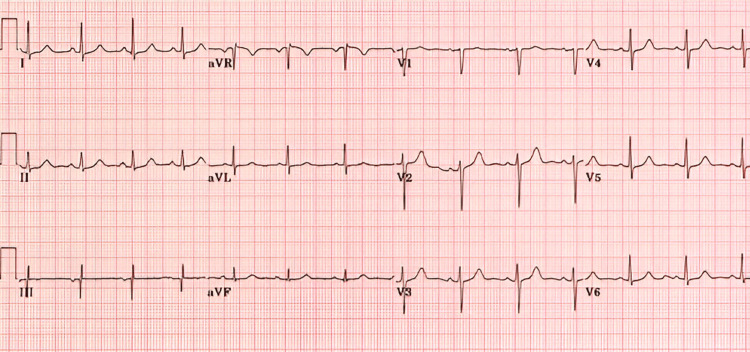
Twelve-lead ECG showing normal sinus rhythm, normal intervals (PR 160 ms, QRS 90 ms, QTc 410 ms), normal axis, and no ST-T wave abnormalities.

A comprehensive laboratory evaluation revealed significant metabolic disturbances and coagulation abnormalities associated with chronic starvation. Blood tests indicated normocytic anemia, with a hemoglobin level of 8.9 g/dL, suggesting a combination of iron deficiency and anemia of chronic disease. Inflammatory markers were elevated, with an ESR of 40 mm/hr and a CRP level of 12 mg/L, indicating a chronic low-grade inflammatory state. Serum albumin was critically low at 2.1 g/dL, confirming severe protein-calorie malnutrition. Electrolyte abnormalities included mild hypokalemia (3.1 mmol/L) (Table 1). Kidney and liver function tests were within normal limits. An ECG was performed and demonstrated no abnormal findings (Figure 1).

**Table 1:** Laboratory investigations demonstrating the patient's abnormal values compared to normal reference ranges.

Lab Test	Patient's Value	Reference Range
Hemoglobin (Hb)	8.9 g/dL	12.0-15.5 g/dL (women)
ESR	40 mm/hr	0-20 mm/hr
CRP	12 mg/L	<5 mg/L
Serum Albumin	2.1 g/dL	3.5-5.5 g/dL
Potassium (K⁺)	3.1 mmol/L	3.5-5.0 mmol/L
Protein C	Normal	70-140% activity
Protein S	Normal	60-130% activity
Antithrombin III	Normal	80-120% activity
Carcinoembryonic Antigen (CEA)	Normal	<3.0 ng/mL
CA-125	Normal	<35 U/mL

**Figure 2:**
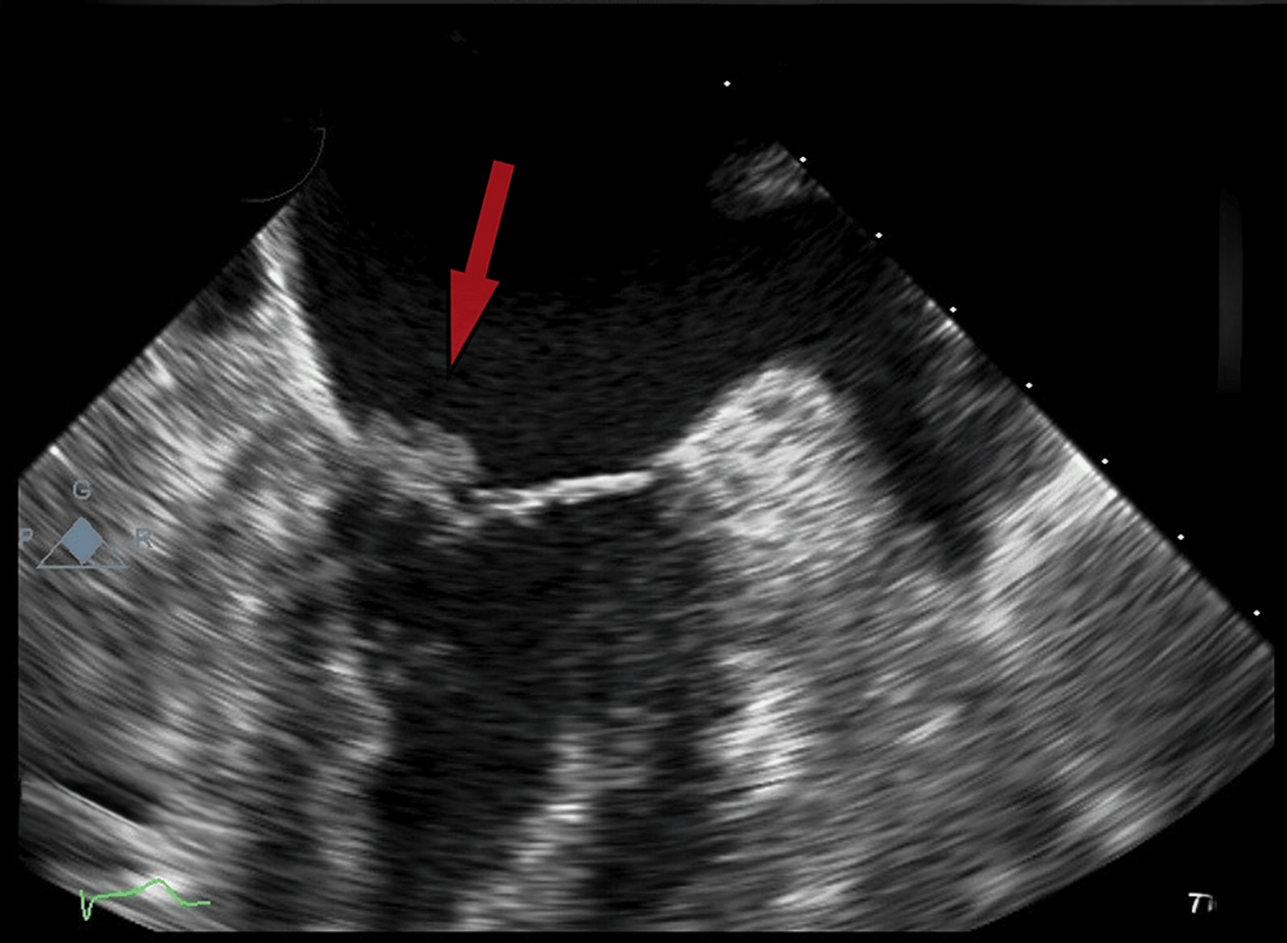
Transthoracic echocardiogram showing vegetations on the anterior mitral valve leaflet (arrow).

**Figure 3:**
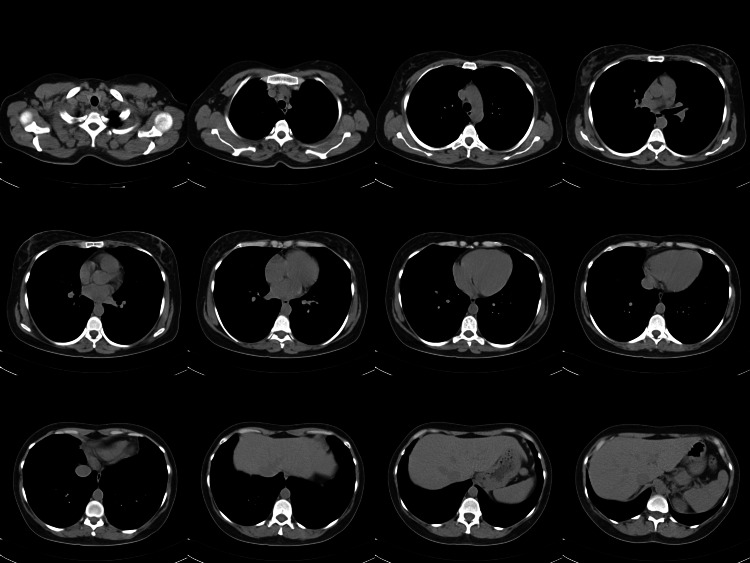
CT scan of the chest showing no remarkable findings in the lungs, mediastinum, or pleural spaces. No radiological evidence of malignancy.

**Figure 4:**
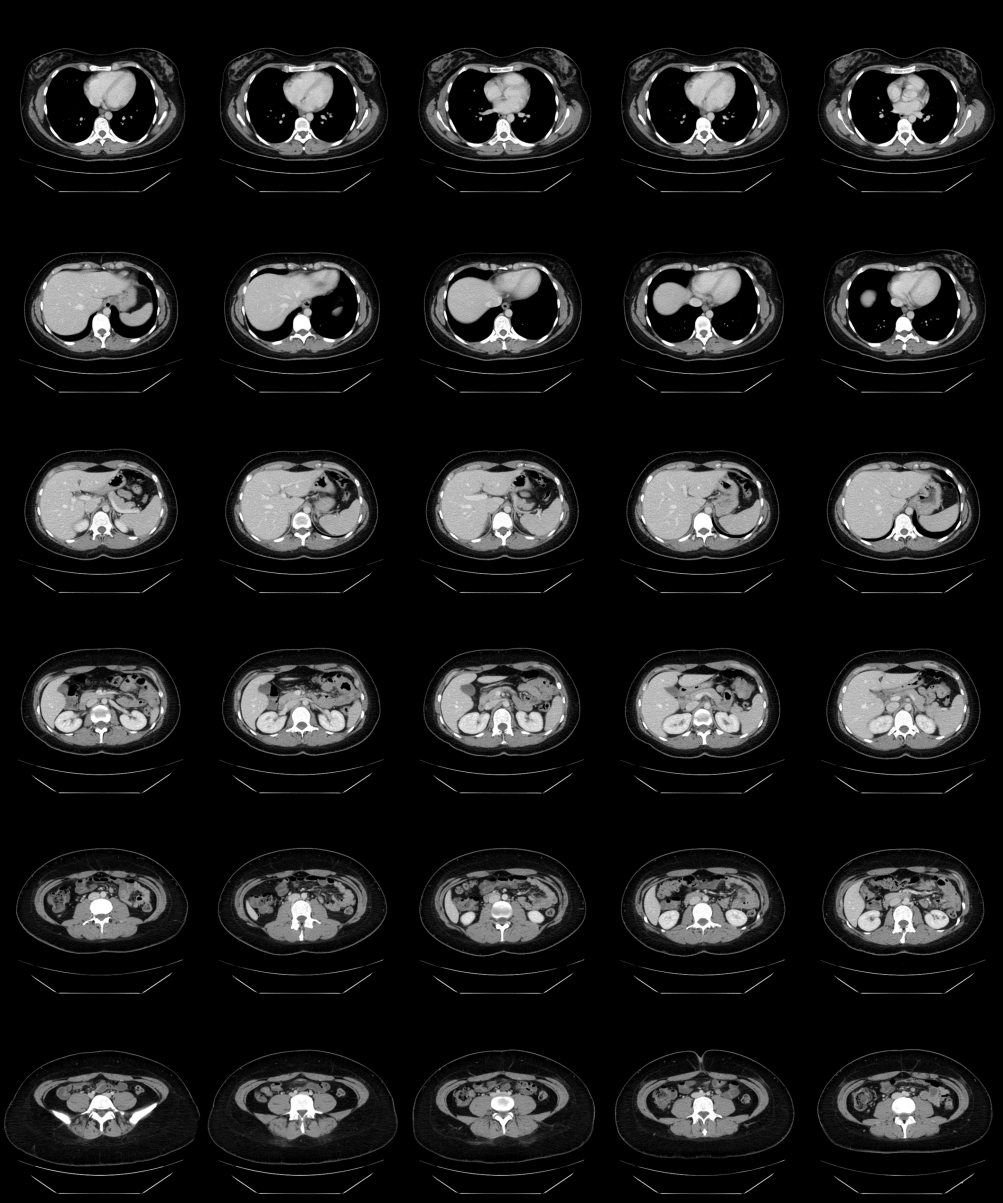
CT scan of the abdomen and pelvis showing no remarkable intra-abdominal or pelvic findings. No evidence of malignancy.

A transthoracic echocardiogram (TTE) (Figure 2) revealed small, mobile, echodense vegetations on the atrial surface of the mitral valve. There was no evidence of valvular dysfunction, abscess formation, or perforation. Three sets of blood cultures remained negative after five days, effectively ruling out infective endocarditis. Further investigations were conducted to identify potential underlying causes. Autoimmune screening, including tests for antiphospholipid antibodies, antinuclear antibodies (ANA), anti-dsDNA, and rheumatoid factor, was negative. Coagulation studies showed normal protein C, protein S, and antithrombin III levels, despite the presence of hypoalbuminemia. Malignancy screening, including chest (Figure 3) and abdominal CT scans (Figure 4), was negative for tumors, and tumor markers such as carcinoembryonic antigen (CEA) and CA-125 (Table 1) were within normal limits. With infections, autoimmune conditions, and cancer ruled out, it seemed most likely that the patient was suffering from NBTE. 

Treatment plan

The treatment strategy was aimed at two primary goals: preventing embolic complications through anticoagulation and initiating nutritional and psychiatric rehabilitation to address the underlying disorder.

Anticoagulation therapy was initiated, with low-molecular-weight heparin (LMWH) administered at weight-adjusted dosing [3]. Warfarin was avoided initially due to the patient’s inconsistent nutritional intake, which could have affected vitamin K-dependent clotting factors [4]. Throughout the hospitalization, the patient was closely monitored for bleeding complications, but no adverse effects were observed.

Nutritional rehabilitation followed a structured refeeding plan to prevent refeeding syndrome [5]. The initial caloric intake was set at 1,200 kcal/day, with gradual increases of 200-300 kcal every 2-3 days. Electrolyte levels were meticulously monitored and supplemented as needed, particularly potassium, magnesium, and phosphate. Thiamine supplementation was administered prophylactically to prevent Wernicke’s encephalopathy [6]. Protein supplementation was incorporated to support albumin production. By week four, the patient’s caloric intake had increased to 2,500 kcal/day, and she had gained 4.5 kg, raising her BMI from 12.5 to 14.2.

Psychiatric and behavioral interventions included enhanced cognitive behavioral therapy (CBT-E) for eating disorders [7], motivational interviewing techniques to enhance treatment adherence, and family therapy sessions [8] to engage her parents in supportive care. No new psychiatric medications were introduced during her hospital stay.

Outcomes and follow-up

**Figure 5:**
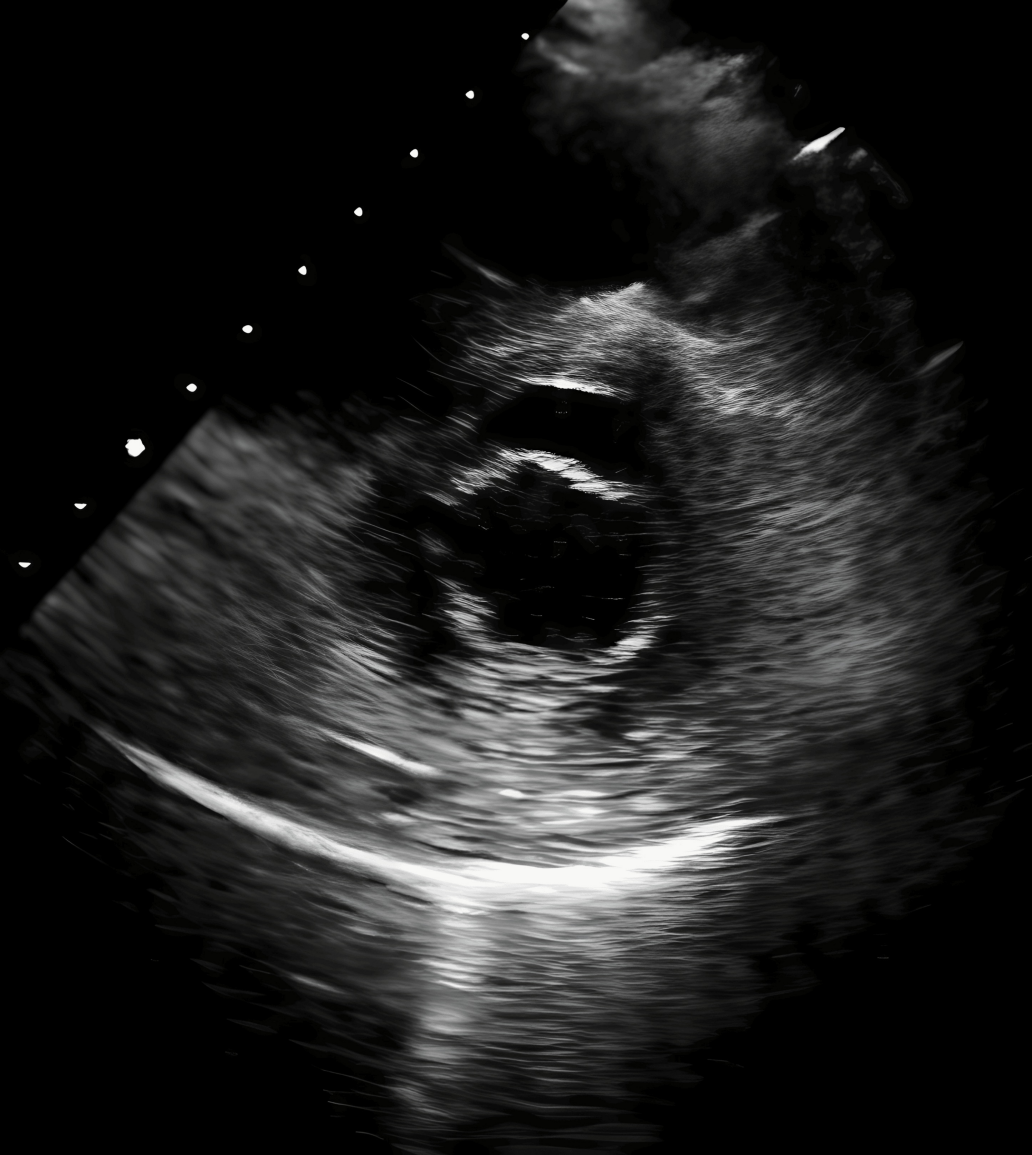
Resolution of vegetations on the mitral valve.

The patient was hospitalized for four weeks, showing substantial clinical improvement. Follow-up echocardiography after four weeks demonstrated complete resolution of the mitral vegetations (Figure 5). Her cardiac status stabilized, with no embolic events reported, a reduction in heart rate from 110 bpm to 80 bpm, and improvement in blood pressure. Hematologic parameters showed an increase in hemoglobin to 10.5 g/dL and serum albumin to 2.8 g/dL. Inflammatory markers also improved, with CRP declining to 5 mg/L. The patient demonstrated significant psychological progress, gaining insight into her condition and showing motivation for continued recovery.

## Discussion

While NBTE is classically associated with advanced malignancies and autoimmune conditions such as systemic lupus erythematosus or antiphospholipid syndrome, its occurrence in the context of malnutrition remains rarely reported. Most available literature describes NBTE in cancer cachexia, where systemic inflammation, endothelial injury, and hypercoagulability form the pathophysiological triad [9]. Similar mechanisms appear to operate in chronic malnutrition, as observed in our patient with anorexia nervosa.

Eiken PW et al. examined 30 surgical pathology specimens of NBTE and emphasized that most patients had underlying malignancies [10]. None had malnutrition as a primary cause. Our case challenges this pattern, suggesting that severe protein-calorie malnutrition alone, without concurrent cancer or autoimmunity, can create a prothrombotic state sufficient to cause NBTE.

Moreover, studies on hypoalbuminemia and thrombosis have shown that low serum albumin independently increases the risk of venous thromboembolism, likely due to impaired hepatic synthesis of anticoagulant proteins and altered endothelial function [11]. This is directly relevant in anorexia nervosa, where hypoalbuminemia is common and often severe.

Finally, Mehler PS and Brown C reviewed cardiovascular complications of anorexia nervosa and noted that while arrhythmias and bradycardia are well-established, valvular lesions are less recognized [12]. Our case adds to this limited body of evidence by demonstrating mitral valve vegetations in the absence of infection, highlighting NBTE as a potentially underdiagnosed cardiac complication in this population.

## Conclusions

This case demonstrates NBTE secondary to severe protein-calorie malnutrition in anorexia nervosa, expanding the etiologic spectrum beyond malignancy and autoimmunity. After systematic exclusion of infective, autoimmune, and neoplastic causes, anticoagulation and enteral nutritional rehabilitation led to complete resolution of valvular vegetations. This report is unique in highlighting starvation alone as a trigger for NBTE and underscores the necessity of multidisciplinary management, including psychiatry, cardiology, hematology, and nutrition teams, to prevent severe complications.
